# Deep generative model of RNAs based on variational autoencoder with context-free grammar

**DOI:** 10.1093/bioinformatics/btaf427

**Published:** 2025-07-29

**Authors:** Goro Terai, Kiyoshi Asai

**Affiliations:** Department of Computational Biology and Medical Sciences, Graduate School of Frontier Sciences, University of Tokyo, Chiba 277-8561, Japan; Department of Computational Biology and Medical Sciences, Graduate School of Frontier Sciences, University of Tokyo, Chiba 277-8561, Japan

## Abstract

**Motivation:**

RNA plays a crucial role in cellular functions, and designing functional RNA sequences is essential for both scientific exploration and bioengineering applications. Conventional RNA design approaches typically assume a shared secondary structure among designed sequences. However, even closely related RNAs can adopt different secondary structures, particularly when artificial mutations are introduced.

**Results:**

We present a novel deep generative model that integrates context-free grammar (CFG) with a variational autoencoder (VAE) to generate RNA sequences while explicitly considering their individual secondary structures. In our method, RNA sequences and their structures are represented as parse trees based on CFG, which are then transformed into binary matrices for VAE training. The optimal parse tree is reconstructed using dynamic programming, ensuring structure-aware sequence generation. When evaluated on natural RNAs from the Rfam database, our model successfully generates high-quality RNA sequences. Furthermore, when applied to RNA aptazyme mutants with distinct secondary structures, our method reveals a strong correlation between the latent space representation of the VAE and self-cleaving activity. This underscores the importance of incorporating RNA-specific structural information in generative models.

**Availability and implementation:**

https://github.com/gterai/RNAgg (archived at Zenodo: https://doi.org/10.5281/zenodo.15354990).

## 1 Introduction

RNA secondary structures function as genetic switches for regulating gene expression (Serganov and Nudler 2013), as components for RNA–protein complex (Reichow *et al.* 2007, Nikulin 2018), as binding sites for proteins (Li *et al.* 2014), and as self-catalytic enzymes (Doherty and Doudna 2000), playing a role in various biological phenomena. Therefore, the ability to design RNA sequences with specific structures and functions is important not only for gaining a deeper understanding of certain biological processes, but also for controlling them.

RNA design has traditionally been approached as an inverse folding problem (Churkin *et al.* 2018). However, inverse folding algorithms focus on designing sequences that adopt a specific secondary structure, making them less suitable for considering properties such as sequence motifs and evolutionary conservation of natural RNAs. Recently, deep generative models (DGMs) have advanced rapidly and have gained significant attention for their ability to generate realistic images and text. DGMs have already been applied successfully to generating protein sequences (Watson *et al.* 2023) and chemical compounds (Gómez-Bombarelli *et al.* 2018). However, current DGMs struggle to accurately account for RNA secondary structures, limiting their suitability for RNA design.

A new RNA design algorithm based on DGMs, called RfamGen, has recently been introduced (Sumi *et al.* 2024). This algorithm can efficiently design novel RNA sequences that belong to a given RNA family and has been experimentally verified to generate RNA that exhibits functions similar, or sometimes surprisingly superior, to those of natural sequences, highlighting the potential of DGM-based RNA design. However, RfamGen assumes that the input RNA sequences share a common secondary structure, which limits its applicability. DGMs have also been used recently to generate diverse RNA sequences likely to adopt a specified tertiary structure (Runge *et al.* 2019, Tan *et al.* 2024, Wong *et al.* 2024), and some of the sequences have had their functionality experimentally verified (Wong *et al.* 2024).

In recent years, studies have been conducted to comprehensively measure the activity of artificially generated RNA mutants using high-throughput sequencing (Kobori and Yokobayashi 2016, Li *et al.* 2016, Kobori *et al.* 2017). Such data may have great potential in the generation of highly active RNA sequences. Since the secondary structures of artificial RNA mutants can differ significantly, a framework is required that can account for these structural changes in order to analyze the data of artificial RNA mutants.

In this study, we propose a novel RNA generation algorithm that combines context-free grammar (CFG) with DGM. Since it does not assume that the training data share a common secondary structure, it is possible to use RNA sequences with structural changes, such as those caused by artificial mutations, as training data. Additionally, since our method can take both aligned and unaligned RNA sequences as input, it allows the incorporation of evolutionary (alignment) information as needed. To evaluate the effectiveness of our method, we generated RNA sequences using natural RNAs from the Rfam database as training data and found that our method produced RNA sequences of similar quality to the training data. Furthermore, when secondary structures of RNA aptazyme mutants were used as training data, mutants with similar activity levels are positioned closer together in the latent space of the variational autoencoder (VAE). A well-organized latent space makes it easier to sample highly active RNA from specific regions within the space.

## 2 Materials and methods

**Figure 1.**
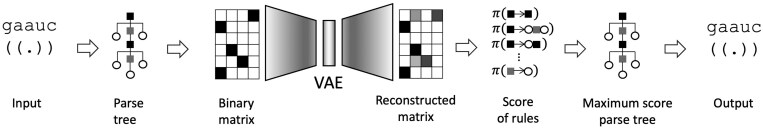
Workflow of the proposed method. The input sequence and structure are transformed into a parse tree with a CFG, encoded into a binary matrix, and processed through a VAE. The reconstructed matrix is used to calculate the scores of production rules of the CFG for obtaining the maximum score parse tree, which represents a generated sequence and structure.

Figure 1 shows an overview of the proposed method. We first represent the RNA sequence and its secondary structure as a parse tree using a CFG. The information in this parse tree is converted into a binary matrix, and a VAE is trained to reconstruct this binary matrix. When generating RNA, the optimal parse tree is obtained from the reconstructed binary matrix using dynamic programming.

### 2.1 CFG used in this study

We use the modified version of the G4 grammar proposed in Dowell and Eddy (2004). The original G4 is as follows:


$$\begin{array}{c} S\to nS|T|\varepsilon \\ T\to Tn|nS\hat{n}|\mathrm{TnS}\hat{n} \end{array}$$

where *T* and *S* are non-terminal symbols, *n* is a terminal symbol (a nucleotide, in this case), $n\hat{n}$ is one of six types of base pairs (*au*, *ua*, *gc*, *cg*, *gu*, *ug*), and ε is an empty symbol.

First, we replaced $nS\hat{n}$ with a new non-terminal *U* and added the rule $U\to nS\hat{n}$. Next, we assigned a pair of indices such as *i* and *j* to all non-terminal symbols. The modified version of the G4 grammar we used in this study is as follows.


$$\begin{array}{c} ss:S_{i,j}\to nS_{i+1,j}\\ st:S_{i,j}\to T_{i,j}\\ tt:T_{i,j}\to T_{i,j-1}n\\ tu:T_{i,j}\to U_{i,j}\\ tb:T_{i,j}\to T_{i,k}U_{k+1,j}\\ us:U_{i,j}\to nS_{i+1,j-1}\hat{n} \end{array}$$

Here, the two letters before the colon indicate the *rule type*, and *i* and *j* are indices representing a pair of positions in the sequence. The reason for using different non-terminal symbols for each pair of positions is to assign distinct *scores* to the production rules corresponding to those positions. These scores are used during a dynamic programming algorithm to obtain the maximum score parse tree, which is explained in more detail later. The above grammar does not contain the output of the empty symbol ε. To compensate for this, when i=j, we used the following production rule: $ss:S_{i,i}\to n$.

### 2.2 Approximate grammar encoding

**Figure 2.**
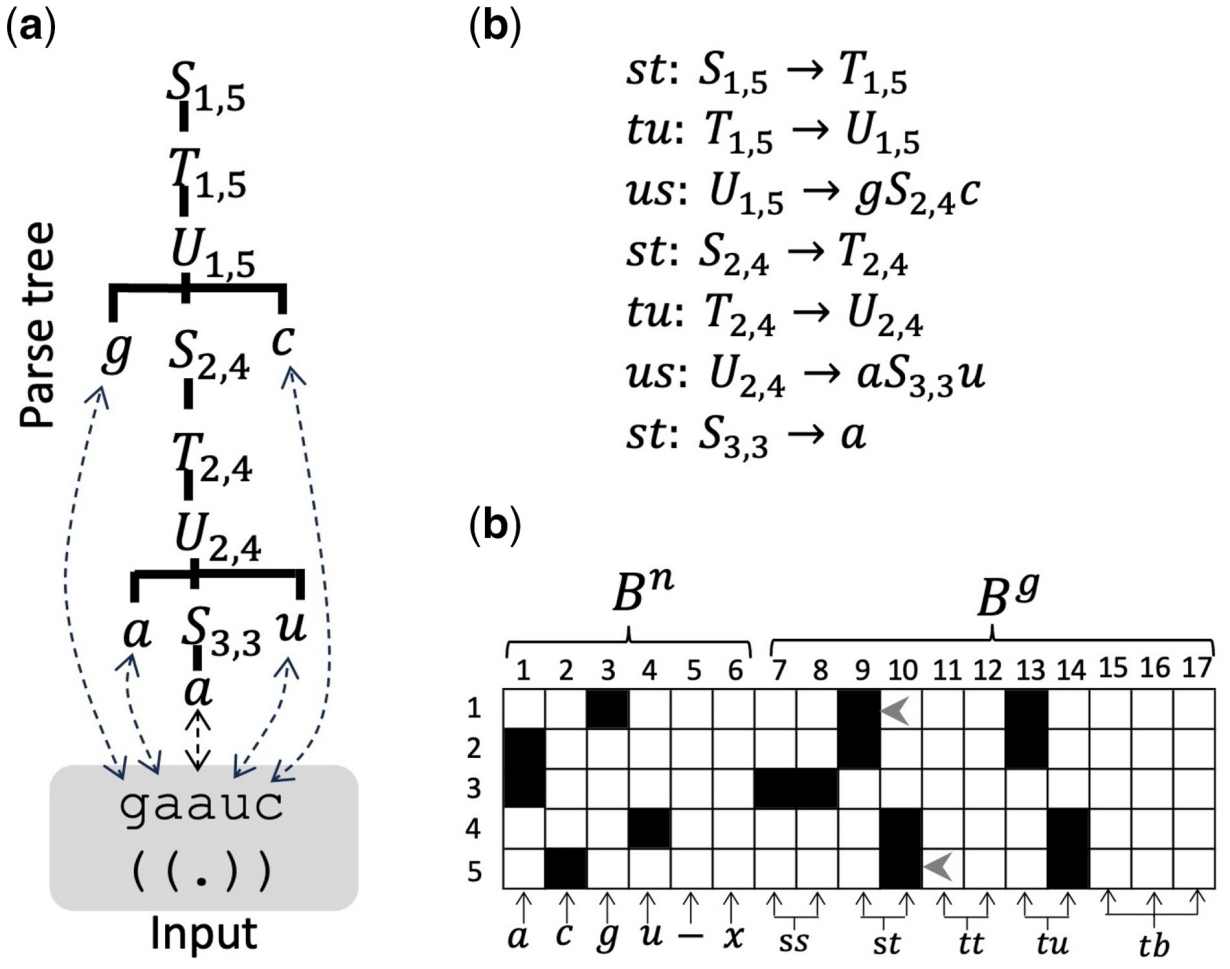
Example of a parse tree and binary matrix. (a) The input sequence and structure (bottom, shaded gray) and corresponding parse tree (top, no shading). Corresponding nucleotides in the sequence and tree are connected by dotted arrows. (b) The set of production rules contained in the parse tree. (c) The binary matrix generated from the rules in (b). White squares represent 0, and black squares represent 1. The rows correspond to nucleotide positions, while the columns correspond to nucleotides (a,c,g,u,−,x) and types of rules (*ss*, *st*, *tt*, *tu*, *tb*). The matrix can be divided into two parts $B^{n}$ and $B^{g}$, each of which play different roles. Arrow heads indicate how the information of the rule $S_{1,5}\to T_{1,5}$ is encoded. For more details, see the main text.

Figure 2a shows an example input and corresponding parse tree. Since the original G4 grammar is unambiguous and always parses the input into exactly one parse tree, the modified version described above is also unambiguous. The parse tree consists of seven types of production rules as described in Fig. 2b. We create a binary matrix from these rules. Figure 2c shows the binary matrix constructed based on these rules. The rows of the binary matrix represent positions, while the columns represent nucleotides (*a*, *c*, …, *x*) and rule types (*ss*, *st*, …, *tb*). The binary matrix can be divided into two parts. Columns 1–6 encode one-hot vectors of the “nucleotides” in each position, and the remaining columns represent the information regarding the “grammar”. We denote the former as $B^{n}$ and the latter as $B^{g}$. For example, $B^{n}$ in Fig. 2c represents the input sequence “gaauc”, expressed as one-hot vectors. The “–” in the fifth column of $B^{n}$ indicates a gap in the alignment and is used when analyzing aligned sequences. The “*x*” in the sixth column is a padding character and is used when analyzing unaligned sequences of different lengths.

Next, we describe how the remaining part of *B*, namely, $B^{g}$, is constructed. The first production rule in Fig. 2b, $S_{1,5}\to T_{1,5}$, is recorded in the 9th and 10th columns because the rule type is *st*. In this production rule, since (*i*, *j*) = (1, 5), the rows (positions) 1 and 5 in the 9th and 10th columns are set to 1, respectively (see arrow heads in Fig. 2c). More formally, this can be described as follows: The binary matrix *B* is initialized with all zeros. The rule types *ss*, *st*, *tt*, and *tu* contain two indices *i* and *j*. When encoding these rule types, we set $B_{i,t}=1$ and $B_{j,t+1}=1$, where *t* is 7, 9, 11, and 13 for *ss*, *st*, *tt*, and *tu*, respectively. The rule type *tb*, which includes *i*, *j*, and *k*, is encoded as $B_{i,15}=1$, $B_{j,16}=1$, and $B_{k,17}=1$.

### 2.3 Maximum score decoding

In this step, the score π for each production rule is calculated from the reconstructed matrix. The parse tree with the maximum score is determined using π by dynamic programming. Below, a production rule is represented in an abbreviated form using the rule type, subscripts, and output nucleotides. For example, the rule $S_{i,j}\to nS_{i,j}$ belongs to rule type *ss* and is written as $ss_{i,j,n}$. Similarly, $S_{i,j}\to T_{i,j}$ is written as $st_{i,j}$. The score π for all production rules is defined below.


$$\begin{array}{c} \pi (ss_{i,j,n})={\hat{B}}_{i,7}\times {\hat{B}}_{j,8}\times {\hat{B}}_{i,I_{n}}\\ \pi (st_{i,j})={\hat{B}}_{i,9}\times {\hat{B}}_{j,10}\\ \pi (tt_{i,j,n})={\hat{B}}_{i,11}\times {\hat{B}}_{j,12}\times {\hat{B}}_{j,I_{n}}\\ \pi (tu_{i,j})={\hat{B}}_{i,13}\times {\hat{B}}_{j,14}\\ \pi (tb_{i,j,k})={\hat{B}}_{i,15}\times {\hat{B}}_{j,16}\times {\hat{B}}_{k,17}\\ \pi (us_{i,j,n,\hat{n}})={\hat{B}}_{i,I_{n}}\times {\hat{B}}_{j,I_{\hat{n}}} \end{array}$$

where $\hat{B}$ represents the reconstructed matrix and $I_{n}$ represents the column of the matrix corresponding to nucleotide *n*. Specifically, $I_{a}$, $I_{c}$, $I_{g}$, $I_{u}$, $I_{-}$, and $I_{x}$ correspond to 1, 2, 3, 4, 5, and 6, respectively. Since the values in $\hat{B}$ are 0–1, all the scores represented by π(·) are also 0–1. Since the associated scores are not normalized as probabilities, our grammar is not strictly a stochastic generative grammar. However, it can be converted into a stochastic CFG (SCFG) by dividing by the partition function for each non-terminal symbol. Thus, our grammar can be regarded as a SCFG in a broader sense.

Next, the optimal parse tree is determined. In this process, three matrices, namely, $M^{S}$, $M^{T}$, and $M^{U}$, of size L×L are used, where *L* is the sequence length. If the input sequence has a variable length, padding letter *x* is added to the end of the sequence, making all sequences the same length. The following recurrence relations are then used to fill in the matrix elements.


$$\begin{array}{c} M_{i,j}^{S}=\max[\begin{array}{c} M_{i+1,j}^{S}\cdot \underset{n\in N}{\max}\{\pi (ss_{i,j,n})\}\\ M_{i,j}^{T}\cdot \pi (st_{i,j}) \end{array}\\ M_{i,j}^{T}=\max[\begin{array}{c} M_{i,j-1}^{T}\cdot \underset{n\in N}{\max}\{\pi (tt_{i,j,n})\}\\ M_{i,j}^{U}\cdot \pi (tu_{i,j})\\ \underset{i<k<j}{\max}\{M_{i,k}^{T}\cdot M_{k+1,j}^{U}\cdot \pi (tb_{i,j,k})\} \end{array}\\ M_{i,j}^{U}=\underset{n\hat{n}\in P}{\max}\;\left[\begin{array}{c} M_{i+1,j-1}^{S}\cdot \pi (us_{i,j,n,\hat{n}}) \end{array}\right] \end{array}$$

where N∈{a,c,g,u,−,x} and P∈{au,ua,cg,gc,gu,ug}, and $M_{1,L}^{S}$ represents the maximum score of the optimal parse tree. The optimal parse tree can be obtained by tracing the matrix $M^{S}$ from 1, *L* across the diagonal elements. In practice, some modifications are introduced mainly to speed up the recursion. A full and exact description of the algorithm is given in the Supplementary Methods, available as supplementary data at *Bioinformatics* online.

### 2.4 Architecture of VAE

We adopted a three-layer fully connected perceptron as the encoder and decoder. The first, second, and third layers of the encoder consist of 4096, 2048, and 516 units, respectively, and the decoder has an architecture that is symmetrical to the encoder (Fig. 1a, available as supplementary data at *Bioinformatics* online). The input and output of the VAE is an L×17-dimensional vector formed by concatenating the rows of the binary matrix. The encoder maps this vector to an eight-dimensional latent space, and the decoder reconstructs the original binary matrix from this latent vector. In the last step of reconstructing the binary matrix, we use softmax and sigmoid functions so that the values in the reconstructed matrix are 0–1 (see, Fig. 1a, available as supplementary data at *Bioinformatics* online for more details).

The loss function for training the VAE is defined as follows. As defined earlier, $B^{n}$ and $B^{g}$ are the 1st–6th and the remaining columns of *B*, respectively.


(1) $$L(\hat{B},\phi ;B)=\mathrm{Rec}(B,\hat{B})+\lambda KL(q_{\phi }(Z|B)∥p(Z))$$

$$\mathrm{Rec}(B,\hat{B})=\alpha CE(B^{n},\hat{B^{n}})+\beta BC(B^{g},\hat{B^{g}})$$

Here, $\hat{B}$ represents the reconstructed *B*, and KL(a||b) is the Kullback–Leibler divergence between distributions *a* and *b*. *Z* is the latent vector of *B*, $q_{\phi }(Z|B)$ is the distribution of *Z* given *B*, and p(Z) is the prior distribution of *Z*, which is assumed to follow a normal distribution. λ is a weight that adjusts the balance between the two terms and is set to λ = 0.001 in this study. CE(N,M) and BC(N,M) represent the cross entropy and binary cross entropy between two matrices, respectively, and α and β are scaling factors used to normalize *CE* and *BC* with respect to the size of *B*. The factor α is defined as 1/L, where *L* is the number of one-hot vectors in $\hat{B^{n}}$, while β is defined as 1/(L·c), where L·c represents the number of elements in $\hat{B^{g}}$. For training the VAE, the Adam optimizer was used with a learning rate of 0.001, the number of epochs was set to 2000, and the batch size was set to 100.

### 2.5 Training data on natural RNA

We trained the VAE using natural RNA sequences and their secondary structures from Rfam release 14.10 (Kalvari *et al.* 2021). Specifically, we selected 10 Rfam families (RF00001 to RF0010), and used the seed sequences and their secondary structures from each family to train the VAE. Positions forming base pairs other than *au*, *ua*, *gc*, *cg*, *gu*, and *ug* were treated as unpaired.

In cases where unaligned RNA was used as training data, we removed all gaps from the alignment. Afterward, padding letter *x* was inserted at the 3′ end of each RNA, and the lengths of all RNAs were standardized to *L*, where *L* is the maximum length of RNA within a particular family.

RNA sequences containing bases other than *a*, *c*, *g*, and *u* and those that could not be parsed using the G4 grammar were excluded from the training data. If the same sequence appeared multiple times, it was included in the training data only once. The number of training data ranged from 61 to 952 depending on the family. Table 1, available as supplementary data at *Bioinformatics* online shows summary of the training data, including the value of *L* and the total number of training sequences.

### 2.6 RNA generation and quality evaluation

When generating RNA, we randomly sampled 1000 points from the latent space according to a normal distribution. For each sampled point, we used the VAE decoder to reconstruct a binary matrix. From the matrix, we generated the maximum score parse tree, which represents the RNA sequence and structure.

We used the same criterion adopted by RfamGen (Sumi *et al.* 2024) to evaluate the quality of generated RNAs. Specifically, the quality of the RNAs was assessed using the bit score from the covariance model in Rfam, which is calculated by the cmalign tool (Nawrocki and Eddy 2013). The bit score can be used as an indicator to determine whether a given sequence belongs to a particular family.

### 2.7 Training VAE considering RNA activity

To incorporate RNA activity into the VAE, we added a multi-layer perceptron for activity prediction to the latent vector of the original VAE (Fig. 1b, available as supplementary data at *Bioinformatics* online). The new loss function combined the original VAE loss (Equation (1)) with the mean squared error for RNA activity values. Hyperparameters, such as the learning rate and number of epochs, were the same as those used in the original VAE.

We used RNA sequence and activity data from aptazymes (Kobori *et al.* 2017), ribozymes (Kobori and Yokobayashi 2016), and tRNA mutants (Li *et al.* 2016). The dataset of aptazymes and ribozymes was obtained from the supplementary website of the respective articles, and the tRNA dataset was obtained from https://github.com/lichuan199010/tRNA_Science. Since these datasets lacked secondary structure information, we predicted RNA secondary structures using mxfold2 (Sato *et al.* 2021) and used these predictions as part of the training data. Activity values were normalized into the range of 0 to 1 for each dataset. Since the RNA mutants in the above studies have the same sequence length within each study, no gaps or padding letters were included in the analysis of these data.

## 3 Results

### 3.1 Generation and evaluation of natural RNA sequences

We trained the proposed VAE using 10 Rfam families and generated RNA sequences for each family. After generating the sequences, we applied filtering to remove those that matched the RNAs in the training data. We also eliminated any duplicate sequences. As a result, about 10% of the generated RNAs were excluded. In the case of the RF0008 family, however, approximately 40% of the RNAs were removed, mainly because they matched sequences from the training data (see Table 2, available as supplementary data at *Bioinformatics* online for details). To evaluate the quality of the generated RNAs, we used the bit scores from the covariance model following the method described by Sumi *et al.* (2024). For comparison, we trained the VAE using aligned and unaligned RNA sequences, which are referred to as RNAgg-ali and RNAgg-una, respectively. Additionally, we trained the VAE with one-hot encoded nucleotide vectors that exclude RNA structure information. In this setup, we also used both aligned and unaligned RNA sequences, referred to as nuc-ali and nuc-una, respectively.

**Figure 3.**
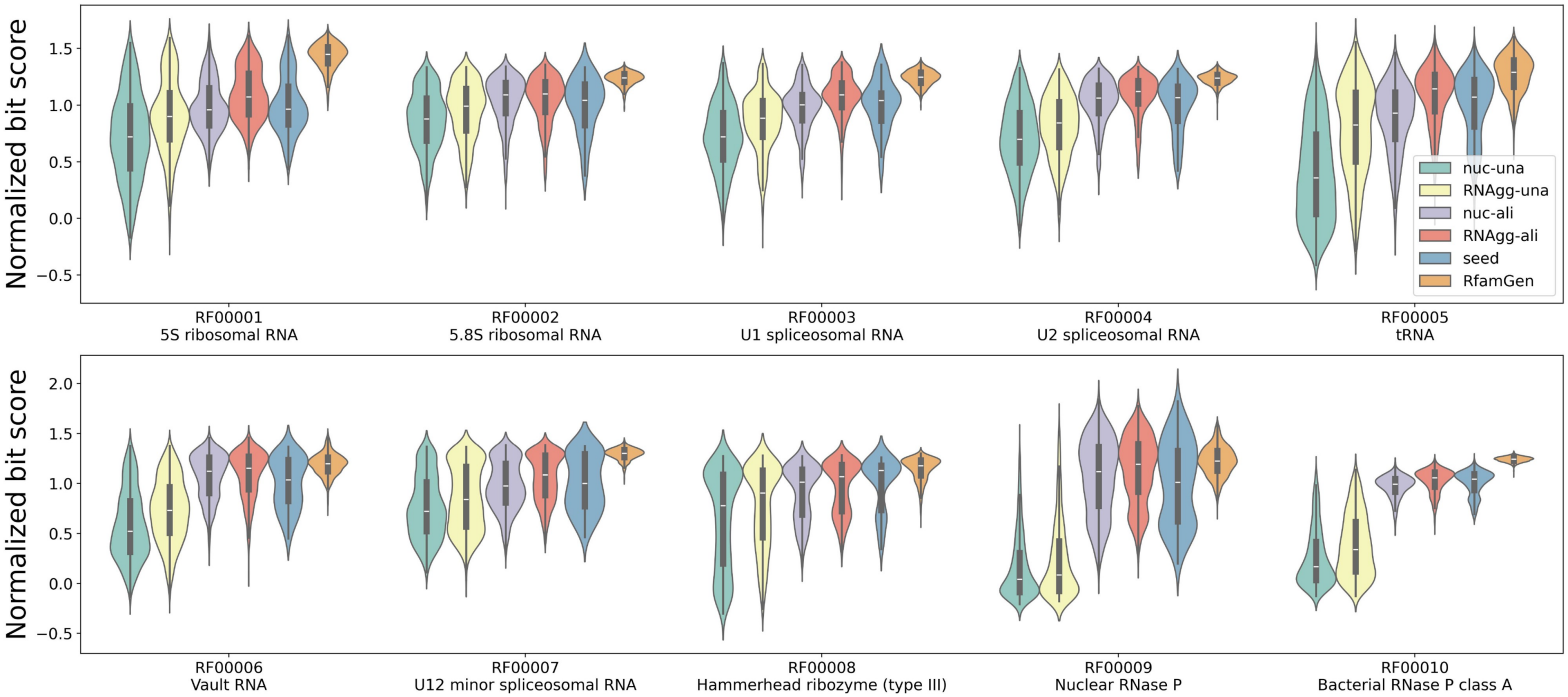
Normalized bit score of generated RNAs for 10 RNA families. The bit scores are normalized so that the mean bit scores of Rfam seed sequences is 1.

Figure 3 shows the distribution of bit scores for generated RNA sequences. For comparison, we also present the bit score distributions of the Rfam seed sequences used as training data and RNA sequences generated using RfamGen (Sumi *et al.* 2024). Across all families, the median bit scores follow the order: nuc-una < RNAgg-una < nuc-ali < RNAgg-ali, with the scores increasing in this order. This indicates that both alignment and secondary structure enhance the generation of higher-quality RNAs. The degree of this trend varies among families and is more pronounced in RF00005, RF00006, RF00009, and RF0010. This suggests that the contributions of structure and alignment differ depending on the RNA family. Interestingly, the median bit scores of RNA sequences generated by RNAgg-ali exceed those of the Rfam seed sequences in all the families expect RF0008, although the difference is slight in some families. Additionally, RfamGen has the highest median bit scores among all families. Thus, when using bit score as an evaluation criterion, RNAgg-ali can generate RNA sequences with bit scores comparable to or higher than the training data. Furthermore, RfamGen generates RNA sequences with even higher bit scores.

Next, we evaluated the diversity of the generated RNA sequences. First, we calculated the pairwise nucleotide identity distribution for the generated RNA sequences. Specifically, we used the cmalign program to align the sequences and calculated the nucleotide identity without counting gaps. The RNA sequences generated by RNAgg-ali had a median pairwise identity of 0.48 to 0.71 depending on the families, while those generated by RfamGen had a higher median pairwise identity of 0.57 to 0.81 (Fig. 2, available as supplementary data at *Bioinformatics* online). Moreover, we found that RNA sequences generated by RfamGen had a notable feature; in most families, the RNA sequences had highly uniform lengths within each family. In contrast, the lengths of RNA sequences generated by our method varied more and were similar to the training data (Fig. 3, available as supplementary data at *Bioinformatics* online). Therefore, the RNA sequences generated by our method had greater diversity in both pairwise nucleotide identity and sequence length compared to those generated by RfamGen.

**Figure 4.**
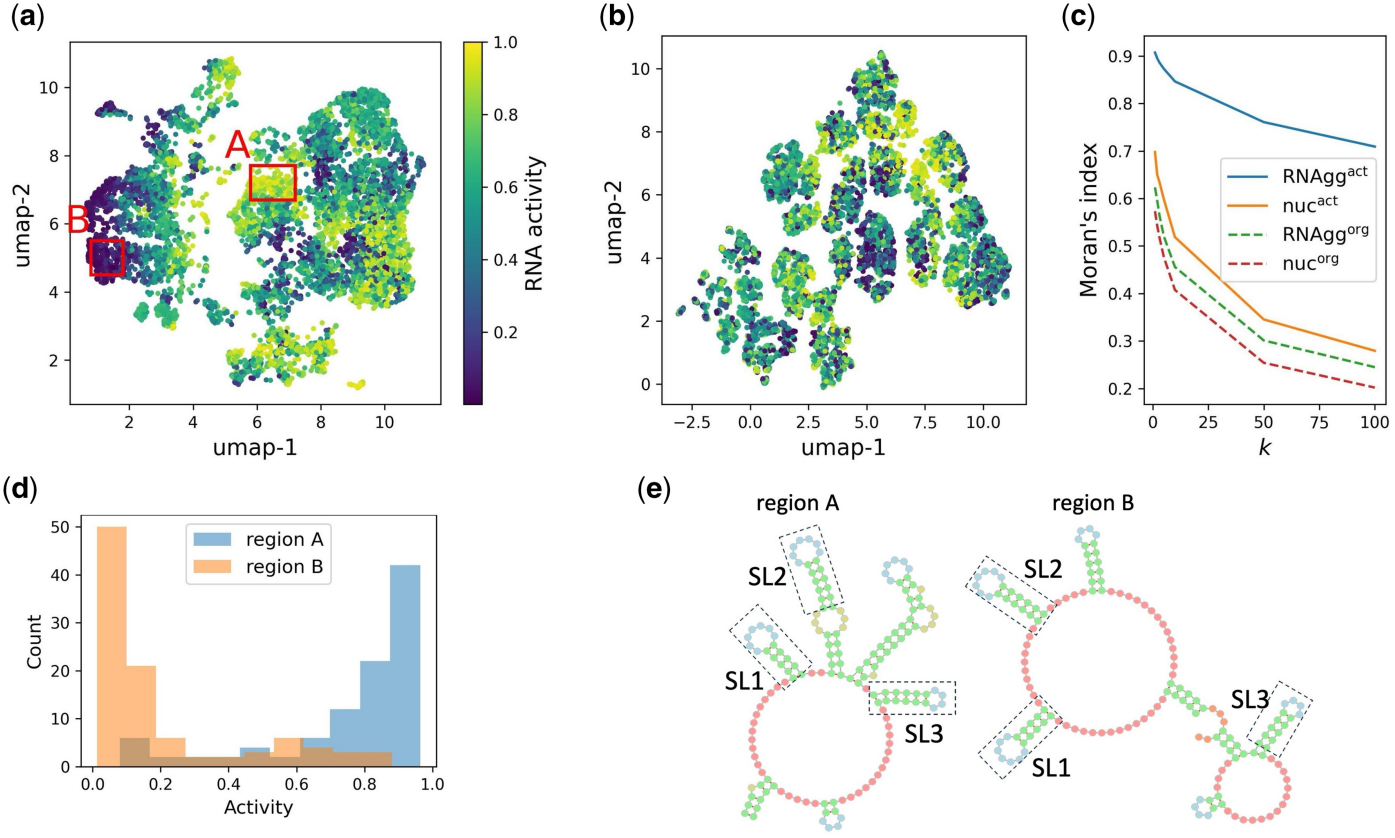
Relationship between aptazyme and self-cleavage activity in latent space. (a) UMAP visualization of aptazymes in the latent space generated by the $\mathrm{RNAg}g^{\mathrm{act}}$ model. The color scale indicates the self-cleavage activity of each aptazyme. (b) UMAP visualization of aptazymes in the latent space generated by the $nuc^{\mathrm{act}}$ model, with the same color scale as in (a). (c) Moran’s I for various methods, showing how activity values cluster spatially. The *x*-axis represents *k*, which is the number of neighbors included in the calculation. (d) Distributions of self-cleavage activity values for aptazymes generated from regions A and B, as highlighted in (a). (e) Comparison of the consensus secondary structures of aptazymes generated from regions A and B. Dashed boxes highlight common stem-loops.

Next, we examined the similarity between the generated RNA sequences and the training data. We calculated the maximum nucleotide identity (Max-NI) between a generated RNA sequence and the training data. Figure 4, available as supplementary data at *Bioinformatics* online shows the distribution of Max-NI values. Whereas the median Max-NI values of the RNA sequences generated by RNAgg-ali were 0.84–0.94 depending on the families, RfamGen generated RNA sequences with median Max-NI values of 0.63–0.91. The median Max-NI values for all generated RNAs were 0.89 and 0.78 for RNAgg-ali and RfamGen, respectively. Thus, our method generated RNA sequences about 11% more identical to the training data than RfamGen on average.

Finally, we analyzed the relationship between bit scores and Max-NI. In general, RNA sequences that are more similar to the training data are expected to have higher bit scores because covariance models are built using the training data. However, for RNA sequences generated by RNAgg-ali, this relationship was less clear compared with those generated by nuc-ali, nuc-una, or RNAgg-una (Fig. 5, available as supplementary data at *Bioinformatics* online). This means that RNA sequences generated by RNAgg-ali tend to have similar bit scores regardless of their nucleotide identity to the training data. Therefore, by considering both alignments and secondary structure, our method makes it easier to generate RNA sequences that differ from the training data but still have high bit scores.

In addition, we trained and evaluated smaller-scale models in which the number of units in the fully connected layers was reduced to 1/16–1/64 of the original. These models were able to generate sequences with comparable bit scores and diversity. Therefore, the parameter size of the model shown in Fig. 1, available as supplementary data at *Bioinformatics* online is likely excessive for the Rfam dataset. However, it remains unclear whether such small-scale models would perform well when the training dataset becomes substantially larger.

### 3.2 Learning curves

Our model contains a large number of parameters, approximately estimated as (17 × *L* + 2048) × 4096 × 2, where *L* is the sequence length. Consequently, for RNA families with sequence lengths exceeding 1000, the number of parameters can reach up to $10^{8}$. To evaluate whether such a highly parameterized model can still learn generalizable patterns from a limited amount of training data, we constructed learning curves for both training and test datasets. For each RNA family, we randomly split the sequences into 70% for training and 30% for testing. The loss values on both datasets were recorded and plotted at each epoch. The learning curves of RNAgg-ali model for each family are shown in Fig. 6, available as supplementary data at *Bioinformatics* online. We observed that test loss decreased along with training loss, suggesting that the model successfully learned meaningful features even from relatively small datasets. Furthermore, the learning curves indicate that the loss plateaued around 100 epochs for all families, implying that longer training durations beyond this point may not be necessary.

### 3.3 RNA reconstruction under noisy grammar encoding

We conducted a sensitivity analysis by introducing random noise into the output of the CFG parser and quantitatively evaluating its effects. Specifically, we injected 10%, 20%, and 30% bit-flip noise into the grammar-related portion of the binary matrices ($B^{g}$), and assessed the changes in the bit scores of the generated sequences with RNAgg-ali model. The results showed that approximately 10% noise had little effect on model performance, but the degradation in sequence quality became apparent with noise levels of 20% or more. In particular, we observed a 3.5% decrease in bit score with 20% noise and a 9.6% decrease with 30% noise. Considering that no noise was introduced into the nucleotide sequences themselves (that is $B^{n}$), this decline in bit score indicates a substantial drop in the quality of the generated sequences. These findings suggest that our model exhibits a certain level of dependence on the accuracy of the CFG parser output.

### 3.4 Generating functional RNAs considering activity

VAE is a highly flexible framework that can incorporate additional features, such as RNA activity, during training. Including RNA activity may help create a more structured latent space, enabling the efficient generation of high-activity RNAs.

We modified the original VAE to incorporate RNA activity (see Fig. 1b, available as supplementary data at *Bioinformatics* online), naming it ${\text{RNAgg}}^{\text{act}}$. For comparison, the same VAE was trained using only nucleotide information, which was referred to as ${\text{nuc}}^{\text{act}}$. Additionally, the original VAE which does not consider RNA activity was trained both with and without RNA secondary structure information, referred to as ${\text{RNAgg}}^{\text{org}}$ and ${\text{nuc}}^{\text{org}}$, respectively.

To evaluate the effect of considering RNA activity, we used the sequence and self-cleavage activity data of aptazyme mutants, as studied by Kobori *et al.* (2017). This dataset contains $4^{7}$ aptazyme mutant sequences that have every possible mutation in a continuous 7-nucleotide sequence near the 5′ end and are all 160 base pairs long. We randomly split the dataset into two halves. One half was used as training data, while the other half was used as test data to evaluate the generation of highly active aptazymes. Figure 4a and b shows the Uniform Manifold Approximation and Projection (UMAP) visualization of the latent spaces produced by ${\text{RNAgg}}^{\text{act}}$ and ${\text{nuc}}^{\text{act}}$, respectively (for comparison, Principal Component Analysis (PCA) visualizations of the same latent spaces are provided in Fig. 7, available as supplementary data at *Bioinformatics* online). The colors represent the activities of the aptazymes. As shown in this figure, aptazymes with similar activities tend to cluster together. To assess this tendency in a quantitative manner, we employed Moran’s index (Moran’s I) (Moran 1950), a statistical measure that evaluates the degree of spatial autocorrelation. Figure 4c compares Moran’s I across four different models. The x-axis *k* represents the number of neighboring aptazymes considered in the calculation of Moran’s I. For instance, when *k *= 1, only the activity with the nearest aptazymes in the 8-dimensional latent space is considered. ${\text{RNAgg}}^{\text{act}}$ have the highest Moran’s I compared to the other models. Therefore, considering the RNA structure and activity makes it easier for aptazymes with similar activities to cluster together. This result suggests that ${\text{RNAgg}}^{\text{act}}$ has learned the relationship between secondary structure and activity, which cannot be captured by nucleotide sequences alone. The Moran’s I values for ${\text{RNAgg}}^{\text{org}}$ and ${\text{nuc}}^{\text{org}}$, which do not consider activity, were lower. This suggests that incorporating RNA activity helps create a more organized latent space in terms of activity.

When the ribozyme and tRNA mutant datasets were used as training data, ${\text{RNAgg}}^{\text{act}}$ and ${\text{nuc}}^{\text{act}}$ achieved higher Moran’s I than ${\text{RNAgg}}^{\text{org}}$ and ${\text{nuc}}^{\text{org}}$. However, differences between ${\text{RNAgg}}^{\text{act}}$ and ${\text{nuc}}^{\text{act}}$ were not observed for these two datasets (Figs 8 and 9, available as supplementary data at *Bioinformatics* online), indicating that incorporating predicted secondary structure did not help create a more structured latent space. We discuss the reason for these results later.

Next, we generated RNA sequences from a cluster of aptazymes with low and high activity, respectively. Specifically, we generated RNA sequences from regions A and B in Fig. 4a. The generation process continued until we obtained 100 distinct sequences matching the test data. Figure 4d compared the activity distribution of the sequences generated from the two regions. As shown, sequences from region A had high activity, while those from region B had low activity. As the difference is expected to be attributed to the RNA secondary structure, we investigated the difference in secondary structures generated from regions A and B. For this purpose, we created the consensus secondary structure of each set of the 100 generated structures (see the Supplementary Methods, available as supplementary data at *Bioinformatics* online). Figure 4e compares the consensus secondary structures of RNAs generated from regions A and B, as depicted by the software Forna (Kerpedjiev *et al.* 2015). These two structures share local similarity, with each having three common stem-loops (dotted boxes in Fig. 4e). The overall structure, however, is clearly different, which likely explains the difference in activity between the sequences from regions A and B.

## 4 Discussion

We proposed an algorithm that combines CFG and VAE to generate RNA sequences while considering the secondary structure of individual RNA. We demonstrated that by using reliable alignment and secondary structure information obtained from the Rfam database, our method can generate RNA with a quality (in terms of bit score) that is comparable to or even higher than that of natural RNA used as training data. While the existing method, RfamGen, generates RNA sequences with higher bit scores than our method, those sequences tend to have uniform lengths and lower nucleotide diversity (higher pairwise nucleotide identity). In contrast, the RNA sequences generated by our method show characteristics, such as length distribution and nucleotide sequences, that are closer to the training data compared with those generated by RfamGen. This suggests that our method and RfamGen explore different search spaces during RNA sequence generation, indicating that the two methods are complementary. It is noteworthy that the RNA generated by our method often has higher bit scores than the training data. As previously discussed in the context of RfamGen, this may be due to the denoising effect of the VAE (Sumi *et al.* 2024). In other words, the generated RNA sequences might not contain unnecessary random mutations, which are present in natural RNA, resulting in higher bit scores.

In our method, we used a modified version of the G4 grammar as the CFG for parsing RNA secondary structures. We selected G4 primarily because it is unambiguous, ensuring that each input sequence maps to a single parse tree. Ambiguous grammars such as G1 or G2 can produce multiple parse trees for the same input, making tree selection non-trivial and potentially inconsistent. Among the available unambiguous grammars, we chose G4 because it produces fewer bifurcations in parse trees—an advantage both for simplifying the binary encoding and for reducing the computational burden of maximum score parsing. Grammars with more bifurcations increase the encoding complexity and slow down parsing, particularly when dealing with long sequences. Therefore, G4 offered a favorable balance between representation simplicity and parsing efficiency in our framework.

The current strategy encodes parse trees into a binary matrix divided into nucleotide information ($B^{n}$) and grammar-related indices ($B^{g}$). Before settling on the current encoding, we experimented with several alternatives. The present approach was ultimately selected for two main reasons: (i) it consistently resulted in high-quality generated sequences, and (ii) it led to more stable training behavior. In this encoding, the positions *i* and *j* involved in base-pairing are encoded independently. For example, a base-pair between positions 1 and 10 is represented by the application of rule $T_{1,10}\to U_{1,10}$, which is encoded by setting $B_{13,1}$ and $B_{14,10}$ to 1. While this representation indicates that position 1 is on the left side of a base-pair, it does not explicitly indicate which position it is paired with. To more precisely represent the i−j pairing information, we explored several alternative encoding schemes. One approach was to use multiple L×L binary matrices, where *L* is the sequence length, in order to encode all i−j pair relationships directly. However, this greatly increased the number of parameters to be learned and resulted in poor performance. We also attempted to encode grammar rules as continuous-valued vectors, where pairs like $B_{13,1}$ and $B_{14,10}$ would have similar values if positions 1 and 10 were paired. Unfortunately, this approach led to unstable training and did not outperform the binary encoding in terms of reconstruction or generation quality. Through these trials and errors, we found that our current binary-vector representation achieved the best balance between expressiveness, training stability, and sequence quality. While it may not perfectly encode all i−j relationships explicitly, it was empirically the most effective among the alternatives we tested.

Our proposed framework adopts a modular design, separating the roles of the VAE and the CFG parser. In this study, we employed a simple VAE composed of a multi-layer fully connected perceptron, primarily because the available training data were limited. In principle, however, the VAE component could be replaced with more powerful generative models such as GANs or diffusion models. As more data become available, or when applying our method to more complex RNA design tasks, it may be appropriate to consider employing such advanced models to improve performance.

The VAE is a versatile tool that can incorporate different features of input data. In this study, we focused on incorporating RNA activity into the training process. As expected, aptazymes with similar activity values tended to be positioned closer to each other in the latent space. Importantly, this tendency was enhanced when secondary structure was considered (Fig. 4c). We hypothesize that the VAE extracted features related to secondary structures directly associated with activity during the training process, positioning aptazymes with these features closer together in the latent space. This tendency was not observed when the ribozyme and tRNA mutant datasets were used as training data. We infer that this is due to the characteristics of the mutant libraries. The aptazyme mutants have every possible mutation in a continuous 7-nucleotide sequence near the 5′ end. Mutations in this local region can influence the secondary structure of the entire RNA, which cannot be fully captured by nucleotide sequence alone. In contrast, ribozyme and tRNA mutants analyzed in this study have mutations spread across a wider region of the RNA. These mutations are more likely to provide enough information for predicting RNA activity, making the consideration of secondary structure less significant. However, it is expected that our method becomes more useful as the accuracy of secondary structure predictions improves. Recent advances in predicting not only the secondary but also tertiary structure may help reduce noise when using predicted structures as training data.

Our method does not assume a common secondary structure across input sequences, which enables it to incorporate a diverse set of RNA families into a unified training dataset. This flexibility opens up the possibility of learning structural or functional representations that are shared across RNA families, potentially leading to more generalizable strategies for RNA design. Moreover, the framework can be readily extended to handle RNA sequences containing modified bases. As computational and experimental techniques for determining RNA secondary structures—including those with modified nucleotides—continue to advance, our model could be adapted to support the design of such RNAs. Furthermore, our VAE-based RNA generator provides a flexible platform for conditional generation. By integrating a pretrained language model that maps natural language descriptions into the latent space, our approach may eventually enable user-guided RNA design driven by textual prompts, such as “self-cleaving aptazyme with high activity”. These features suggest that our model can serve as a versatile foundation for future applications in RNA engineering.

## Supplementary Material

btaf427_Supplementary_Data

## Data Availability

The data underlying this article are publicly available with their sources described in the article. The source code is available at GitHub: https://github.com/gterai/RNAgg. An archived version is available on Zenodo: https://doi.org/10.5281/zenodo.15354990.

## References

[btaf427-B1] Churkin A , Retwitzer MD, Reinharz V et al Design of RNAs: comparing programs for inverse RNA folding. Brief Bioinform 2018;19:350–8. 28049135 10.1093/bib/bbw120 PMC6018860

[btaf427-B2] Doherty EA , Doudna JA. Ribozyme structures and mechanisms. Annu Rev Biochem 2000;69:597–615. 10966470 10.1146/annurev.biochem.69.1.597

[btaf427-B3] Dowell RD , Eddy SR. Evaluation of several lightweight stochastic context-free grammars for RNA secondary structure prediction. BMC Bioinformatics 2004;5:71. 15180907 10.1186/1471-2105-5-71 PMC442121

[btaf427-B4] Gómez-Bombarelli R , Wei JN, Duvenaud D et al Automatic chemical design using a data-driven continuous representation of molecules. ACS Cent Sci 2018;4:268–76. 29532027 10.1021/acscentsci.7b00572 PMC5833007

[btaf427-B5] Kalvari I , Nawrocki EP, Ontiveros-Palacios N. Rfam 14: expanded coverage of metagenomic, viral and microRNA families. Nucleic Acids Res 2021;49:D192–200. 33211869 10.1093/nar/gkaa1047 PMC7779021

[btaf427-B6] Kerpedjiev P , Hammer S, Hofacker IL. Forna (force-directed RNA): simple and effective online RNA secondary structure diagrams. Bioinformatics 2015;31:3377–9. 26099263 10.1093/bioinformatics/btv372 PMC4595900

[btaf427-B7] Kobori S , Yokobayashi Y. High-throughput mutational analysis of a twister ribozyme. Angew Chem Int Ed Engl 2016;55:10354–7. 27461281 10.1002/anie.201605470 PMC5113685

[btaf427-B8] Kobori S , Takahashi K, Yokobayashi Y. Deep sequencing analysis of aptazyme variants based on a pistol ribozyme. ACS Synth Biol 2017;6:1283–8. 28398719 10.1021/acssynbio.7b00057

[btaf427-B9] Li C , Qian W, Maclean CJ et al The fitness landscape of a tRNA gene. Science 2016;352:837–40. 27080104 10.1126/science.aae0568 PMC4894649

[btaf427-B10] Li X , Kazan H, Lipshitz HD et al Finding the target sites of RNA-binding proteins. Wiley Interdiscip Rev RNA 2014;5:111–30. 24217996 10.1002/wrna.1201 PMC4253089

[btaf427-B11] Moran PAP. Notes on continuous stochastic phenomena. Biometrika 1950;37:17–23. 15420245

[btaf427-B12] Nawrocki EP , Eddy SR. Infernal 1.1: 100-fold faster RNA homology searches. Bioinformatics 2013;29:2933–5. 24008419 10.1093/bioinformatics/btt509 PMC3810854

[btaf427-B13] Nikulin AD. Structural aspects of ribosomal RNA recognition by ribosomal proteins. Biochemistry (Mosc) 2018;83:S111–33. 29544435 10.1134/S0006297918140109

[btaf427-B14] Reichow SL , Hamma T, Ferré-D’Amaré AR et al The structure and function of small nucleolar ribonucleoproteins. Nucleic Acids Res 2007;35:1452–64. 17284456 10.1093/nar/gkl1172 PMC1865073

[btaf427-B15] Runge F , Stoll D, Falkner S et al Learning to design RNA. In: *The 7th International Conference on Learning Representations (ICLR2019)*. New Orleans, LA, USA, 2019.

[btaf427-B16] Sato K , Akiyama M, Sakakibara Y. RNA secondary structure prediction using deep learning with thermodynamic integration. Nat Commun 2021;12:941. 33574226 10.1038/s41467-021-21194-4 PMC7878809

[btaf427-B17] Serganov A , Nudler E. A decade of riboswitches. Cell 2013;152:17–24. 23332744 10.1016/j.cell.2012.12.024 PMC4215550

[btaf427-B18] Sumi S , Hamada M, Saito H. Deep generative design of rna family sequences. Nat Methods 2024;21:435–43. 38238559 10.1038/s41592-023-02148-8

[btaf427-B19] Tan C , Zhang Y, Gao Z et al RDesign: Hierarchical data-efficient representation learning for tertiary structure-based RNA design. In: *The Twelfth International Conference on Learning Representations *(ICLR2024)**. New Orleans, LA, USA, 2024.

[btaf427-B20] Watson JL , Juergens D, Bennett NR et al De novo design of protein structure and function with RFdiffusion. Nature 2023;620:1089–100. 37433327 10.1038/s41586-023-06415-8 PMC10468394

[btaf427-B21] Wong F , He D, Krishnan A et al Deep generative design of RNA aptamers using structural predictions. Nat Comput Sci 2024;4:829–39. 10.1038/s43588-024-00720-6 39506080 PMC12743617

