# Supplementary material for: Deep generative model of RNAs based on variational autoencoder with context-free grammar
Source: Bioinformatics. 2025 Jul 29;41(8):btaf427. doi: 10.1093/bioinformatics/btaf427 (PMC12342829; doi:10.1093/bioinformatics/btaf427)
Supplement: btaf427_Supplementary_Data [file btaf427_supplementary_data.pdf]

1 Supplementary Methods

1.1 Obtaining an optimal RNA sequence and structure

The optimal RNA sequence and secondary structure are determined using dynamic programming. In this process, three DP matrices, M^S, M^T, and M^U, of size $L \times L$ are used, where L is the sequence length. First, the three matrices are initialized as follows. For $i = 1$ to L,

$$M^S_{i,i} = \max_{n \in N}\{\pi(ss_{i,i,n})\}$$
$$M^T_{i,i} = M^U_{i,i} = 0$$

where $N \in \{a, c, g, u, -\}$. The following recurrence relations are then used to fill in the matrix elements for $i < j$.

$$M^S_{i,j} = \max \begin{bmatrix} M^S_{i+1,j} \cdot \max_{n \in N}\{\pi(ss_{i,j,n})\} & \text{(Eq. 1)} \\ M^T_{i,j} \cdot \pi(st_{i,j}) & \text{(Eq. 2)} \end{bmatrix}$$

$$M^T_{i,j} = \max \begin{bmatrix} M^T_{i,j-1} \cdot \max_{n \in N'}\{\pi(tt_{i,j,n})\} & \text{(Eq. 3)} \\ M^U_{i,j} \cdot \pi(tu_{i,j}) & \text{(Eq. 4)} \\ \max_{i<k<j}\{M^T_{i,k} \cdot M^U_{k+1,j} \cdot \pi(tb_{i,j,k})\} & \text{(Eq. 5)} \end{bmatrix}$$

$$M^U_{i,j} = \max_{n\hat{n} \in P}\left[M^S_{i+1,j-1} \cdot \pi(us_{i,j,n,\hat{n}})\right] \quad \text{(Eq. 6)}$$

where $N' \in \{a, c, g, u, -, x\}$ and $P \in \{au, ua, cg, gc, gu, ug\}$. Since (Eq. 5) includes a loop over k, the computational cost to calculate each element in M^T is $O(L)$. Therefore, completing this recursive calculation requires $O(L^3)$ total computation. To speed up this calculation and impose some constraints on generated RNAs, we adopted some heuristics as follows.

- To reduce the computation of (Eq. 5), we evaluate this equation only when $\pi(tb_{i,j,k})$ is not small. As described in the main text, $\pi(tb_{i,j,k}) = \hat{B}_{i,15} \times \hat{B}_{j,16} \times \hat{B}_{k,17}$, where $\hat{B}$ is a reconstructed matrix. We evaluate (Eq. 5) only when $\hat{B}_{i,15} \times \hat{B}_{j,16} > 10^{-5}$ and $\hat{B}_{k,17} > 10^{-5}$. In addition, we do not evaluate (Eq. 5) when $\hat{B}_{i,15} \times \hat{B}_{j,16}$ is less than the maximum of (Eq. 3) and (Eq. 4).
- We impose the condition that there are at least 3 nucleotides between a base pair in generated RNA. For this purpose, we set $M^T_{i,j} = M^U_{i,j} = 0$ for cases where $j - i + 1 < 5$. Thus, we do not evaluate (Eq. 5) when $j - i + 1 < 10$. This is because (Eq. 5) is for evaluating subsequences having at least two base pairs, meaning that the subsequences must have a length of 10 or more. Thus, evaluation of (Eq. 5) for subsequences with less than 10 is unnecessary.

1.2 Making the consensus secondary structure

Let W be a set of RNA secondary structures. We assume that all secondary structures in W have the same length. A base pair (i, j) is called a consensus base pair if it forms a pair in more than half of the structures in W. Let C represent the set of all consensus base pairs. If C does not include pseudoknots, it represents a single, pseudoknot-free secondary structure. In this study, since C contained no pseudoknots, we were able to create a single consensus secondary structure that includes all base pairs in C.

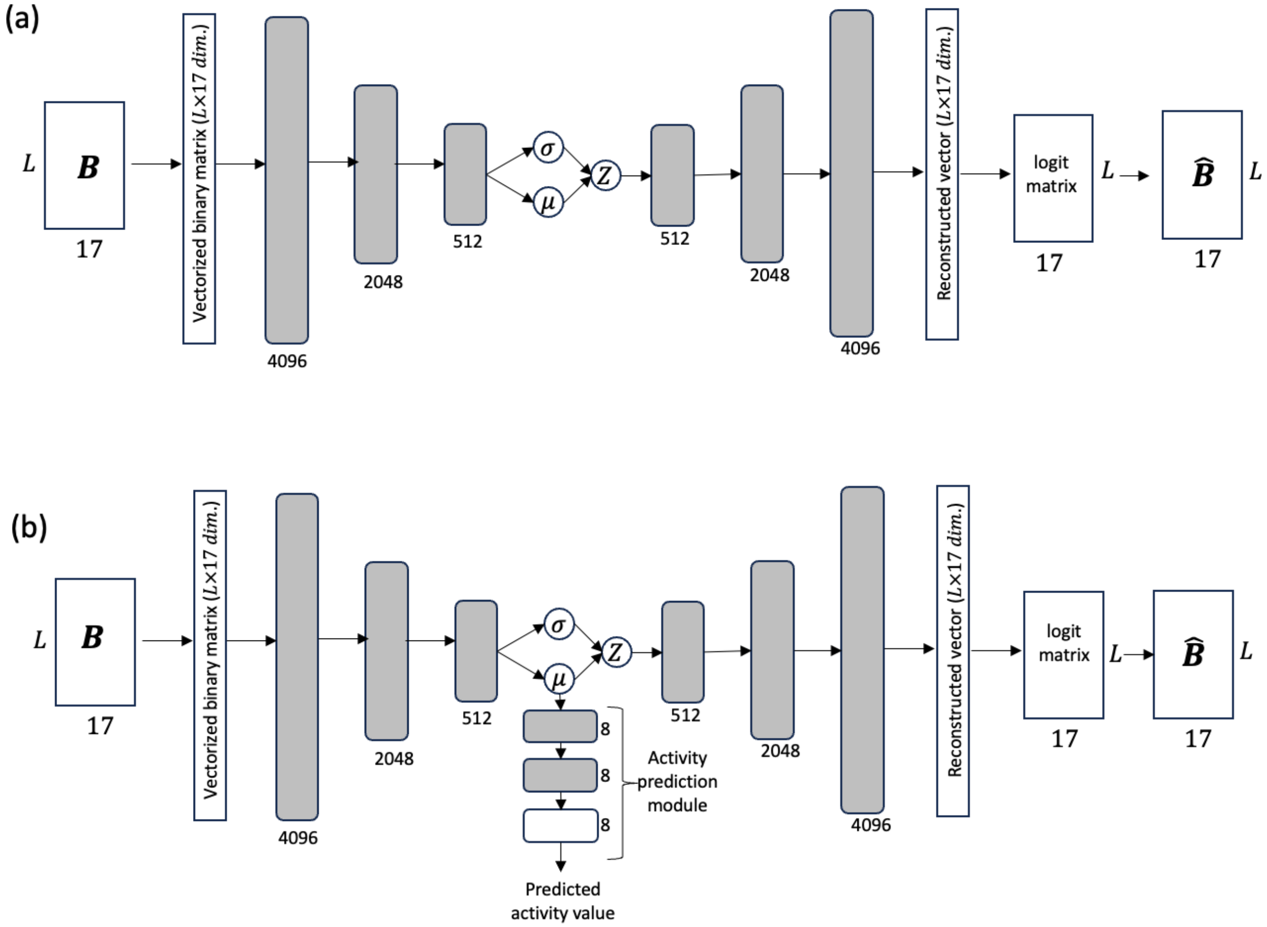

Figure S1: Architecture of VAE used in this study. (a) VAE without activity prediction module. B and $\hat{B}$ are a binary matrix and the reconstructed one, respectively. Rounded gray squares represent fully connected layers, with numbers indicating the units in each layer. Each layer has batch normalization and a ReLU activation function. μ and σ are 8-dimensional mean and standard deviation vectors, respectively, which generate an 8-dimensional latent vector Z. When obtaining $\hat{B}$ from the logit matrix, we applied the softmax function to the 1st to 6th columns of the logit matrix, and the sigmoid function to the remaining columns so that the values in $\hat{B}$ are 0-1. (b) VAE with activity prediction module. This module has three layers, each with 8 units. It takes the 8-dimensional mean vector (μ) as input and predicts activity values based on it. The last layer of the activity prediction module does not use batch normalization or ReLU activation but simply performs a linear summation.

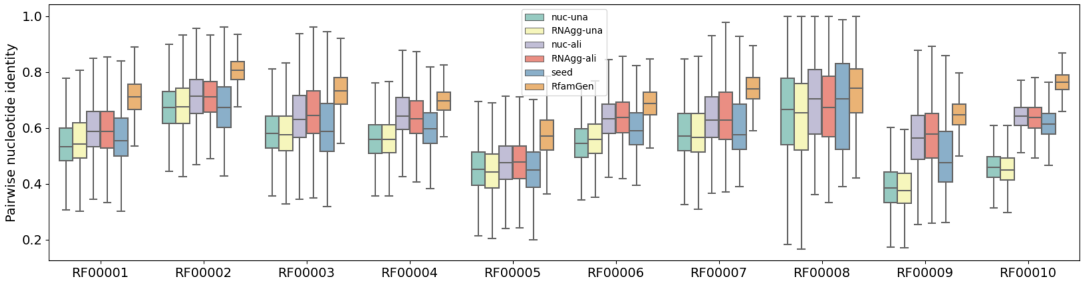

Figure S2: Distribution of pairwise nucleotide identity between generated RNAs. The distribution for seed sequences is also included for comparison.

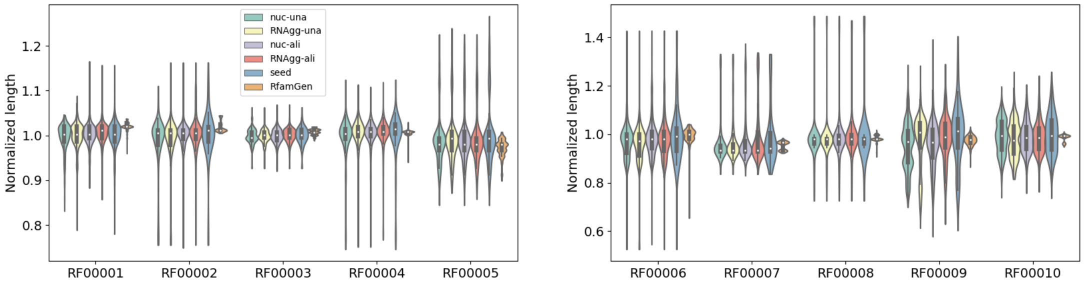

Figure S3: Length distribution of generated RNAs. The length is normalized such that the mean length of the seed sequence is 1. The distribution for seed sequences is also included for comparison.

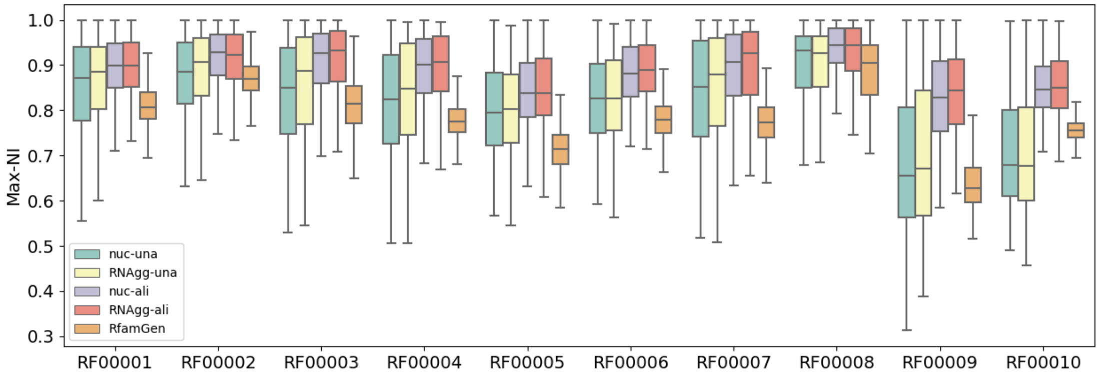

Figure S4: Distribution of maximum nucleotide identity to training data (Max-NI) of generated RNAs.

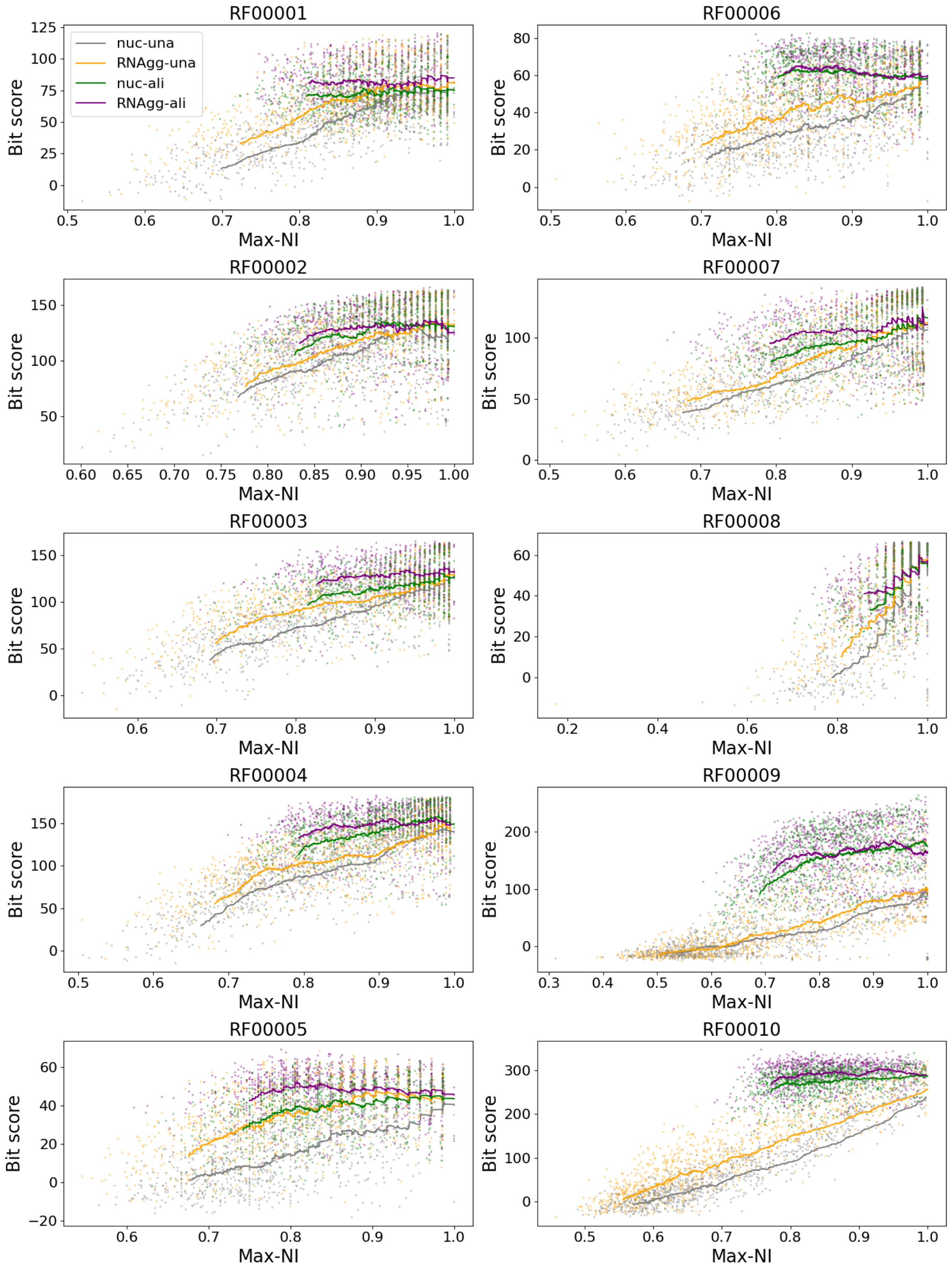

Figure S5: Relationship between bit score and maximum nucleotide identity (Max-NI). Each point represents a generated RNA, and the lines indicate the moving average of bit scores over 100 points, showing how the bit score changes as Max-NI decreases.

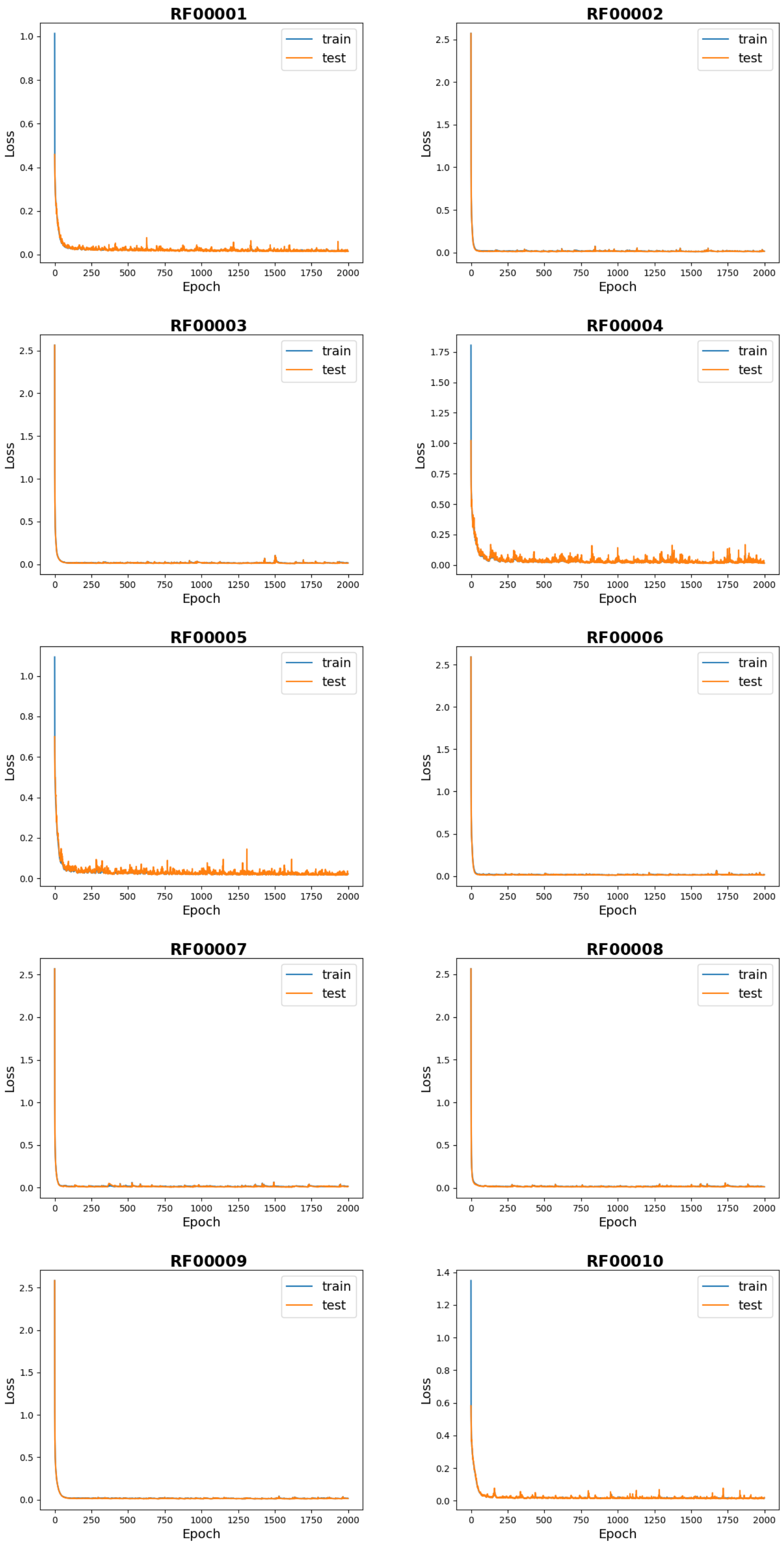

Figure S6: Training curves with train and test error

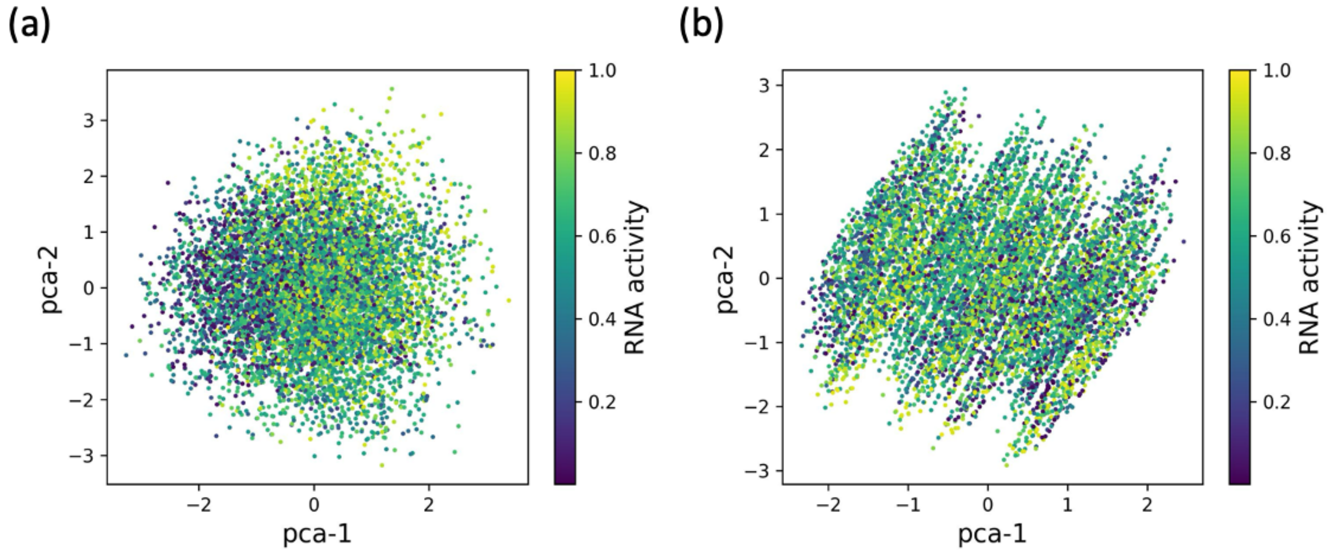

Figure S7: Relationship between aptazyme sequences and self-cleavage activity in the latent space. PCA plots of aptazymes are shown, colored by their self-cleavage activity values for (a) $\mathrm{RNAgg}^{\mathrm{act}}$ and (b) $\mathrm{nuc}^{\mathrm{act}}$. The color scale indicates the level of self-cleavage activity. For plot (a), the explained variance ratios of the first and second principal components were 0.1408 and 0.1354, respectively, while for plot (b), they were 0.1492 and 0.1456. These relatively low variance ratios suggest that the linear transformations applied by PCA are insufficient to capture the complex structure of the latent space learned by our VAE.

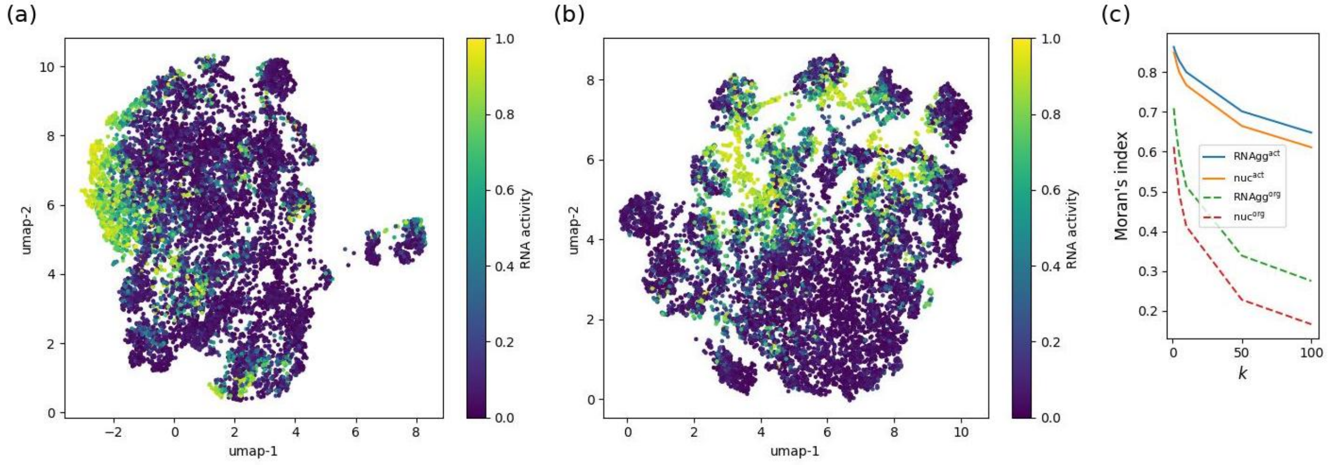

Figure S8: Relationship between twister ribozyme and self-cleavage activity in latent space. UMAP plots of ribozymes colored by their self-cleavage activity values for (a) $\mathrm{RNAgg}^{\mathrm{act}}$ and (b) $\mathrm{nuc}^{\mathrm{act}}$. The color scale represents self-cleavage activity. (c) Moran's index comparison for different methods.

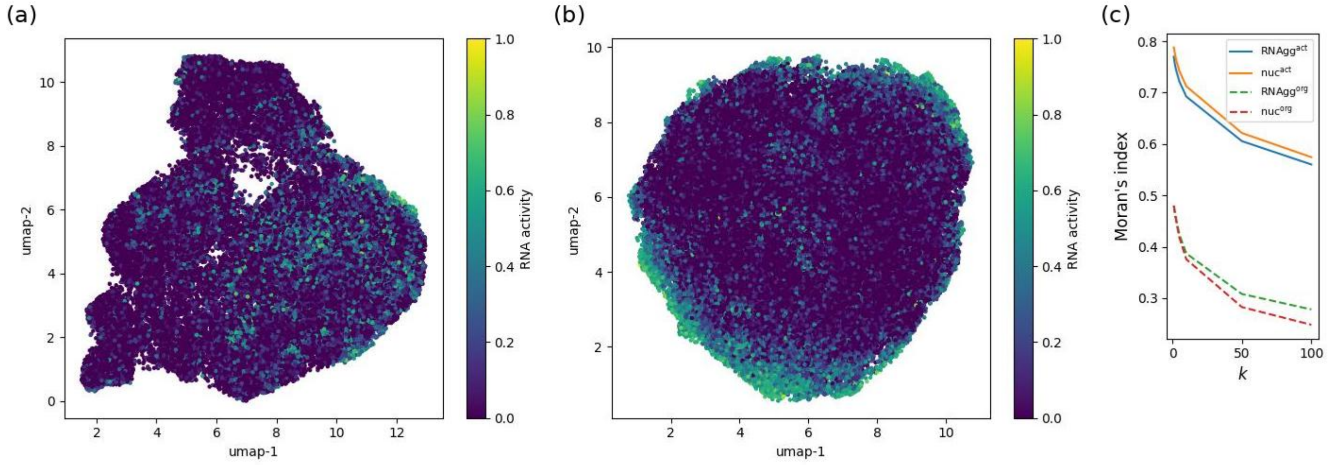

Figure S9: Relationship between tRNA and fitness activity in latent space. UMAP plots of tRNAs colored by their fitness activity values for (a) RNAgg^{act} and (b) nuc^{act}. The color scale represents fitness activity. (c) Moran's index comparison for different methods.

Table S1: Summary of Rfam training data

Rfam ID	Num[a)]	Len[b)]	Ident[c)]	$L^{d)}$	$L_{ali}^{e)}$	Description
RF00001	683	116.7	0.57	135	230	5S rRNA
RF00002	61	152.2	0.68	177	207	5.8S rRNA
RF00003	77	161.8	0.62	172	203	U1 spliceosomal RNA
RF00004	190	190.4	0.61	214	278	U2 spliceosomal RNA
RF00005	952	73.4	0.45	93	118	tRNA
RF00006	72	100.9	0.60	144	163	Vault RNA
RF00007	59	161.6	0.62	215	269	U12 minor spliceosomal RNA
RF00008	85	55.1	0.68	82	85	Hammerhead ribozyme
RF00009	112	313.4	0.51	440	845	Nuclear RNase P
RF00010	410	381.8	0.62	480	996	Bacterial RNase P class A

[a)] Number of sequences used for training
[b)] Mean length of training sequences
[c)] Mean pairwise identity without considering gaps
[d)] Maximum length of unaligned sequences
[e)] Length of aligned sequences

Table S2: Number of generated sequences after filtering

**Model**	**Filtering Step**	**RF00001**	**RF00002**	**RF00003**	**RF00004**	**RF00005**
nuc-una	Remove training data	927	889	898	953	958
	Remove duplication	926	886	891	953	958
RNAgg-una	Remove training data	921	873	872	968	939
	Remove duplication	921	870	867	965	939
nuc-ali	Remove training data	902	870	848	947	941
	Remove duplication	902	859	839	946	941
RNAgg-ali	Remove training data	915	863	823	919	930
	Remove duplication	915	860	816	919	930

**Model**	**Filtering Step**	**RF00006**	**RF00007**	**RF00008**	**RF00009**	**RF00010**
nuc-una	Remove training data	923	836	730	958	991
	Remove duplication	922	820	677	958	991
RNAgg-una	Remove training data	925	870	712	948	994
	Remove duplication	922	860	632	945	994
nuc-ali	Remove training data	875	813	681	978	986
	Remove duplication	874	792	586	978	986
RNAgg-ali	Remove training data	867	825	646	980	987
	Remove duplication	865	806	514	980	987
